# Intracerebral hemorrhage associated with brucellosis: A case report

**DOI:** 10.3389/fneur.2022.1038201

**Published:** 2022-12-22

**Authors:** Yusong Luo, Guopeng Tian, Maoqiang Lin, Xiang Fang, Shengwei Bai, Yawen Pan

**Affiliations:** ^1^Department of Neurosurgery, Lanzhou University Second Hospital, Lanzhou, China; ^2^Department of Orthopedics, Lanzhou University Second Hospital, Lanzhou, China

**Keywords:** brucellosis, intracerebral hemorrhage, vascular walls, mechanisms, diagnosis

## Abstract

**Background:**

Intracerebral hemorrhage is a common disease, but cases of intracerebral hemorrhage with brucellosis are very rare. Here, we are presenting a case of a 60-year-old male patient diagnosed with brucellosis who has a right basal ganglia hemorrhage ruptured into bilateral lateral ventricles.

**Case presentation:**

A 60-year-old male patient with symptoms of intracerebral hemorrhage who had no common risk factors for intracerebral hemorrhage, but having been diagnosed with brucellosis 2 months earlier and telling a shepherd history for 3 years. Cranial computed tomography (CT) and cranial magnetic resonance angiography (MRA) revealed that an intracerebral hemorrhage in the right basal ganglia had broken into bilateral lateral ventricles, and a Brucella serology test was positive. The patient's condition improved significantly after receiving bilateral lateral ventricle cone drainage, hematoma cavity cone drainage and anti-brucellosis treatment.

**Conclusions:**

Herein, we discuss the possible mechanisms and clinical implications between brucellosis and intracerebral hemorrhage. This case suggests whether we can use brucellosis as a routine examination for disease diagnosis and prevention in patients with intracerebral hemorrhage from pastoral areas.

## Introduction

Brucellosis existing worldwide is a zoonotic disease resulting from Brucella species infection (1). Brucellosis in China is primarily concentrated in pastoral areas such as Northwest China. The incidence of brucellosis is closely related to people's eating habits and occupation, for example, people are susceptible to Brucella when they regularly drink or eat unpasteurized dairy products (2, 3). In patients with brucellosis, nervous system involvement is ~4–7% (4). In fact, intracerebral hemorrhage associated with brucellosis is not common, and the pathogenesis remains unclear. Herein, we present a case of a 60-year-old male patient with chronic brucellosis with right basal ganglia hemorrhage breaking into bilateral lateral ventricles. We speculate that intracerebral hemorrhage could be linked to Brucella infection. This article aims to discuss the possible mechanism between brucellosis and intracerebral hemorrhage and to provide some help for clinical diagnosis, treatment and prevention.

## Case report

**Figure 1:**
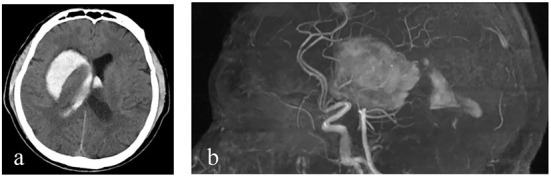
Imaging findings. **(a, b)** Cranial CT and cranial MRA show an intracerebral hemorrhage in the right basal ganglia and in bilateral lateral ventricles.

**Figure 2:**
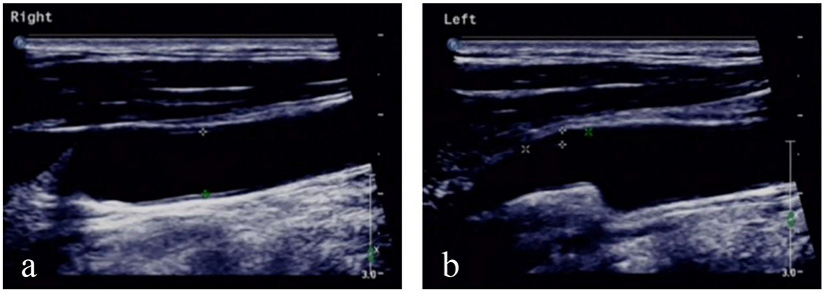
Ultrasound of neck vessels. **(a)** Ultrasound of the right common carotid artery showed a diameter of 0.78 cm and an intima-media thickness of 0.10 cm. **(b)** Left common carotid artery ultrasound showed a diameter of 0.75 cm, an intima-media thickness of 0.10 cm, and a hypoechoic flat plaque (2.00 × 0.37 cm) on the distal posterior wall.

A 60-year-old male patient from a rural area presented to the outpatient clinic of our hospital with fever, night sweats, headache, dizziness, nausea, and vomiting as the chief complaints. In addition, the patient had some symptoms, such as migratory pain in the right hip joint, right knee joint, lower back, and back. With brucellosis being diagnosed 2 months ago, the patient had a 3-year history of herding sheep. The patient had a history of polio for 58 years, which was manifested by muscle atrophy of the right lower extremity. Upon admission, neurological examination indicated that the patient's mouth angle was oblique to the right and that his left nasolabial fold became shallow. The muscular strength examination revealed the strength of muscle was grade 4 in the left upper limb, grade 2 in the left lower limb, grade 5 in the right upper limb, and grade 4 in the right lower limb. The patient's body temperature was 37.5°C, and blood pressure was 125/78 mmHg. The Glasgow coma score (GCS) of the patient was 15 (E4V5M6). CT (Figure 1a) and MRA (Figure 1b) revealed that an intracerebral hemorrhage in the right basal ganglia had broken into bilateral lateral ventricles. Ultrasonography of cervical vessels revealed bilateral carotid medial thickening with plaque formation (Figures 2a,b). A Brucella serology test showed that the Rose Bengal test and Wright agglutination test with a titer of 1/100 were positive. Findings of serological infection markers showed that procalcitonin had increased to 0.219 ng/mL (reference value, 0.000–0.046 ng/mL) and interleukin 6 (IL-6) had increased to 66.26 pg/mL (reference value, 0.00–7.00 pg/mL). Serum biochemical tests found that the level of high-density lipoprotein (HDL) had decreased to 0.75 mmol/L (reference value, 1.03–1.55 mmol/L). Disseminated intravascular coagulation (DIC) examination showed that D-dimer had increased to 7.25 μg/mL (reference value, 0.00–0.50 μg/mL), and fibrinogen degradation products (FDP) had increased to 24.74 μg/mL (reference value, 0.00–5.00 μg/mL).

**Figure 3:**
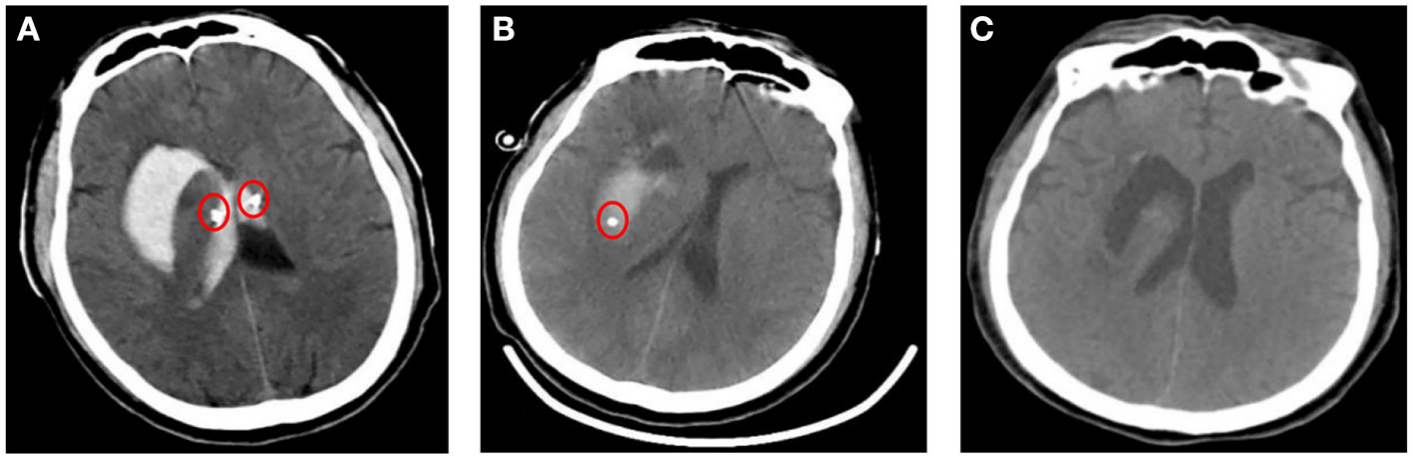
Imaging findings after treatments. **(A, B)** Images of a cranial CT after implantation of a drainage tube (red circle) though drilling in bilateral lateral ventricle and in the right basal ganglia. **(C)** CT after removing the drainage tube showed normal bilateral lateral ventricles and a cavity in right basal ganglia.

**Figure 4:**
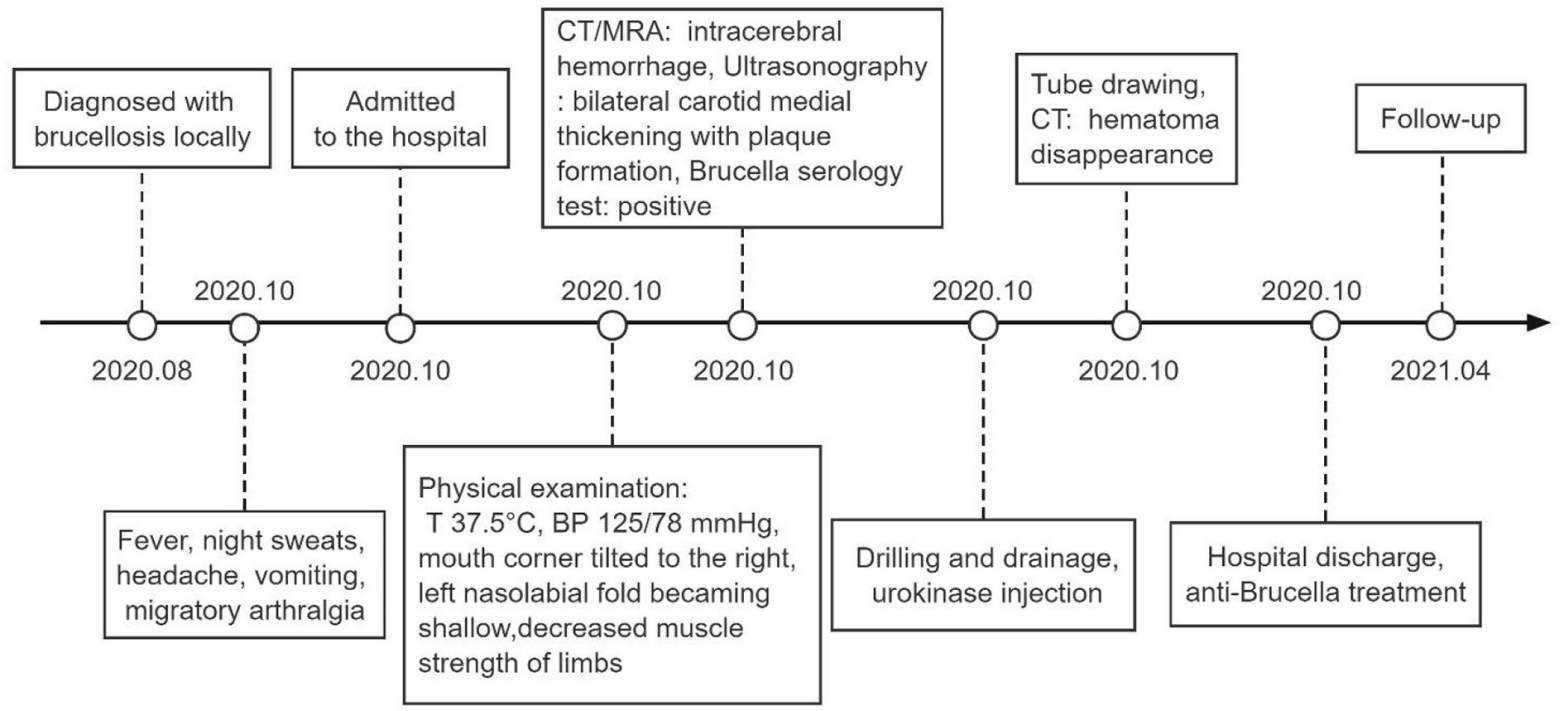
Timeline. T, temperature; BP, blood pressure; CT, computed tomography; MRA, magnetic resonance angiography.

The patient was given bilateral lateral ventricle drilling with ventricular drainage in the emergency department of the intensive care unit. The bilateral puncture points were 2.5 cm next to the midline and 2.5 cm before the coronal suture. Along the line connecting the external auditory canals on both sides, toward the tip of the nose, the cranial cone was inserted into the brain for 2.5 cm, and then the cannula was inserted into the brain for 6.5 cm (Figure 3A). At the same time, 5 million U of urokinase was injected through the drainage tube every 12 h for thrombolytic therapy. After 10 days of the treatment, as the patient's symptoms were relieved, the re-examination of cranial CT showed a significant decrease in the size of the bilateral lateral ventricular hematoma, but the right basal ganglia hematoma still existed. Therefore, the bilateral lateral ventricle drainage tubes were pulled out, and the right basal ganglia hematoma cavity was drained at the same time. The right puncture point was located 2.5 cm beside the midline and 3.0 cm anterior to the coronal suture. Toward the center of the hematoma, the cranial cone was inserted 2.5 cm into the brain, and then the cannula was inserted into the brain 6.5 cm (Figure 3B). After about 7 days of treatment, the patient's symptoms were significantly relieved, and head CT showed that the hematoma disappeared, and the drainage tube was subsequently removed (Figure 3C). The condition of the patient improved significantly after adequate treatment. The muscle strength of the right lower limb was grade 4, and the muscle strength of the other limbs was grade 5. When discharged, the patient was instructed to continue taking oral antiepileptic drugs. In addition, anti-Brucella treatment with oral rifampicin (900 mg/day) for 6 weeks and oral doxycycline (100 mg twice daily) for 45 days was persevered. After follow-up by telephone for 6 months, the patient had no symptoms related to intracerebral hemorrhage or brucellosis (Figure 4).

## Discussion

Intracerebral hemorrhage is a common cause of death and disability and the established risk factors include obesity, high blood pressure, diabetes, smoking, drinking, trauma, aneurysm, and arteriovenous malformation (5). In this case, the patient did not have those associated risk factors, surprisingly, the patient reported that he had been diagnosed with brucellosis in a local hospital, and had suspected brucellosis clinical manifestations such as fever, night sweats and migratory arthralgia. Although blood and cerebrospinal fluid were not cultured for Brucella, combined with the positive Brucella serology tests including Rose Bengal test and Wright agglutination test, the patient could be diagnosed with brucellosis according to CARE guideline (6, 7). Therefore, we highly suspect that intracerebral hemorrhage could be related to brucellosis. During the course of treatment, we gave the patient drilling and drainage to clear intracranial hematoma, mannitol as well as sodium aescinate to reduce intracranial pressure, sodium valproate to prevent epilepsy, cephalosporin to prevent drainage tube infection, respiratory nebulization to expel sputum to prevent lung infection caused by long-term bed rest, dezocine to stop the head temporarily, and physically cooling down to maintain normal body temperature. Although repeated blood and cerebrospinal fluid bacterial culture tests showed no abnormalities in process of treatment, body temperature monitoring showed wavy fever. The patient admitted to have a history of brucellosis when we asked again and the diagnosis of brucellosis was confirmed after reexamination, so the patient was given doxycycline and rifampicin for anti-brucellosis according to CARE guideline (7).

To date, literatures have only been reported for a small number of the intracerebral hemorrhages related to brucellosis, none of which has made the clear explanation of the mechanism (8–10). Here, we propose the following mechanisms of brucellosis possibly promoting intracerebral hemorrhage. Brucellosis can cause damage to the blood vessel wall and atherosclerosis due to inflammation, increased oxidative stress, accumulation of endotoxin, elevation of autoimmune cytokines and lipid accumulation in blood vessel walls (11, 12). Therefore, brucellosis can promote intracerebral artery rupture and hemorrhage under the action of hemodynamics. According to reports, some patients with brucellosis develop thrombocytopenia, and mechanisms of which may include increased clearance, bone marrow suppression, immune-mediated destruction, disseminated intravascular coagulation, dysplasia of bone marrow megakaryocytes, hypersplenism, platelet phagocytosis and adhesion to damaged blood vessel surfaces (13, 14). Meanwhile, reduced or defective coagulation factors of the patient are detected, and mechanisms of which may include the reduction of coagulation factor synthesis or abnormal factor production due to liver damage caused by Brucella as well as activation of fibrinolysis or intravascular activation of coagulation (13). However, the limitation of this report is that the vascular condition decreasing with advancing age could not be excluded. We need more cases and researches to establish a definite causational link between brucellosis and intracerebral hemorrhage.

By information from this case, we speculate that brucellosis may have adverse effects on intracerebral hemorrhage by affecting vascular walls, platelets and coagulation factors. This has a certain guiding significance for clinical diagnosis and treatment, and improves the understanding of the correlation between brucellosis and intracerebral hemorrhage.

## Data availability statement

The raw data supporting the conclusions of this article will be made available by the authors, without undue reservation.

## Ethics statement

Written informed consent was obtained from the individual(s) for the publication of any potentially identifiable images or data included in this article.

## Author contributions

YL and GT: investigation. YL: writing-original draft preparation. YL, ML, and XF: writing-review and editing. YL and SB: visualization. YP: supervision. All authors have read and agreed to the published version of the manuscript.

## References

[1] Gul HC Erdem H Bek S. Overview of neurobrucellosis: a pooled analysis of 187 cases. Int J Infect Dis. (2009) 13:e339–43. 10.1016/j.ijid.2009.02.015 19428283

[2] Herrick JA Lederman RJ Sullivan B Powers JH Palmore TN. Brucella arteritis: clinical manifestations, treatment, and prognosis. Lancet Infect Dis. (2014) 14:520–6. 10.1016/S1473-3099(13)70270-6 24480149 PMC4498663

[3] Majzobi MM Karami P Khodavirdipour A Alikhani MY. Brucellosis in humans with the approach of Brucella species contamination in unpasteurized milk and dairy products from Hamadan, Iran. Iran J Med Microbiol. (2022) 16:2–2. 10.30699/ijmm.16.4.282

[4] Shakir R. Brucellosis. J Neurol Sci. (2021) 420:117280. 10.1016/j.jns.2020.117280 33358192

[5] Li C Ford ES Zhao G Balluz LS Berry JT Mokdad AH. Undertreatment of mental health problems in adults with diagnosed diabetes and serious psychological distress: the behavioral risk factor surveillance system, 2007. Diabetes Care. (2010) 33:1061–4. 10.2337/dc09-1515 20185747 PMC2858175

[6] Ramin B Macpherson P. Human brucellosis. BMJ. (2010) 341:c4545. 10.1136/bmj.c4545 20833741

[7] Franco MP Mulder M Gilman RH Smits HL. Human brucellosis. Lancet Infect Dis. (2007) 7:775–86. 10.1016/S1473-3099(07)70286-4 18045560

[8] Spyrou A Natsis KS Papamichalis E Mourtzinos H. Intraventricular haemorrhage and seizures in a patient with dementia: a case of chronic neurobrucellosis. Age Ageing. (2019) 48:601–2. 10.1093/ageing/afz020 30843580

[9] Korri H Awada A Ali Y Choucair J. Brucellar meningitis complicated by aneurysmal subarachnoid hemorrhage. Rev Neurol. (2008) 164:1052–5. 10.1016/j.neurol.2008.05.001 18808777

[10] Al-Harthi SS. Association of brucella endocarditis with intracerebral haemorrhage. Int J Cardiol. (1987) 16:214–6. 10.1016/0167-5273(87)90256-7 3623729

[11] Apostolou F Gazi IF Kostoula A Tellis CC Tselepis AD Elisaf M. Persistence of an atherogenic lipid profile after treatment of acute infection with Brucella. J Lipid Res. (2009) 50:2532–9. 10.1194/jlr.P900063-JLR200 19535817 PMC2781324

[12] Karahocagil MK Aslan M Ceylan MR Cikman A Sunnetcioglu M Kucukoglu ME. Serum myeloperoxidase activity and oxidative stress in patients with acute brucellosis. Clin Biochem. (2012) 45:733–6. 10.1016/j.clinbiochem.2012.03.017 22465269

[13] Crosby E Llosa L Miro Quesada M Carrillo C Gotuzzo E. Hematologic changes in brucellosis. J Infect Dis. (1984) 150:419–24. 10.1093/infdis/150.3.419 6481187

[14] Pappas G Kitsanou M Christou L Tsianos E. Immune thrombocytopenia attributed to brucellosis and other mechanisms of Brucella-induced thrombocytopenia. Am J Hematol. (2004) 75:139–41. 10.1002/ajh.10473 14978693

